# AR-42 induces apoptosis in human hepatocellular carcinoma cells via HDAC5 inhibition

**DOI:** 10.18632/oncotarget.8077

**Published:** 2016-03-12

**Authors:** Mingming Zhang, Yida Pan, Robert G. Dorfman, Zhaogui Chen, Fuchen Liu, Qian Zhou, Shan Huang, Jun Zhang, Dongqin Yang, Jie Liu

**Affiliations:** ^1^ Department of Digestive Diseases of Huashan Hospital, Fudan University, Shanghai, China; ^2^ Collaborative Innovation Center of Genetics and Development, Institutes of Biomedical Sciences and Department of Immunology, Shanghai Medical School, Fudan University, Shanghai, China; ^3^ Northwestern University Feinberg School of Medicine, Chicago, IL, USA; ^4^ Department of Gastroenterology, Nanjing Drum Tower Hospital, Medical School of Nanjing University, Nanjing, China; ^5^ Department of Hepatobiliary Surgery, The Eastern Hepatobiliary Surgery Hospital of Second Military Medical University, Shanghai, China; ^6^ School of Life Sciences, Fudan University, Shanghai, China; ^7^ Department of Pathology, The Second Hospital of Anhui Medical University, Anhui Medical University, Hefei, China

**Keywords:** AR-42, apoptosis, hepatocellular carcinoma, HDAC5, prognosis

## Abstract

Histone deacetylases (HDACs) play critical roles in apoptosis and contribute to the proliferation of cancer cells. AR-42 is a novel Class I and II HDAC inhibitor that shows cytotoxicity against various human cancer cell lines. The present study aims to identify the target of AR-42 in hepatocellular carcinoma (HCC) as well as evaluate its therapeutic efficacy. We found that HDAC5 was upregulated in HCC tissues compared to adjacent normal tissues, and this was correlated with reduced patient survival. CCK8 and colony-formation assays showed that HDAC5 overexpression promotes proliferation in HCC cell lines. Treatment with AR-42 decreased HCC cell growth and increased caspase-dependent apoptosis, and this was rescued by HDAC5 overexpression. We demonstrated that AR-42 can inhibit the deacetylation activity of HDAC5 and its downstream targets *in vitro* and *in vivo*. Taken together, these results demonstrate for the first time that AR-42 targets HDAC5 and induces apoptosis in human hepatocellular carcinoma cells. AR-42 therefore shows potential as a new drug candidate for HCC therapy.

## INTRODUCTION

As resistance to conventional anti-cancer drugs becomes increasingly commonplace, research aimed at developing new strategies for treating hepatocellular carcinoma (HCC) in the clinic, as well as identifying new tumor markers, is urgently needed [1]. HCC is one of the most common malignant cancers worldwide, causing more than half a million deaths annually and ranking as the third leading cause of cancer-related mortality [2].

The histone deacetylases (HDACs) are enzymes that catalyze the removal of acetyl groups from histones. According to their homology and structure, the 11 HDACs are divided into Classes I, II and IV, whereas the 7 sirtuins make up Class III [3]. Class I HDACs (1, 2, 3 and 8) and Class II HDACs (4, 5, 6, 7, 9 and 10) play an important role in tumorigenesis and may be candidate targets for cancer treatment [4–8]. Numerous reports indicate HDACs are overexpressed in many cancers (especially HDAC1, HDAC2 and HDAC8 in colon cancer) and inhibit specific tumor suppressor genes, resulting in an aberrant epigenetic status compared to adjacent normal cells [9, 10]. HDAC5, a Class II HDAC, has been shown to play a critical role in cell proliferation and apoptosis in different cancers, and HDAC5 expression is increased in liver cancer tissues [11], suggesting that it may be a potential marker of poor patient survival [12–14]. However, the molecular mechanism of HDAC5 in human HCC remains unclear.

Because acetylation-mediated epigenetic changes are reversible, HDAC inhibitors show potential as promising chemotherapeutic agents. Indeed, numerous studies reported that HDAC inhibitors (Vorinostat (Suberoylanilide Hydroxamic Acid; SAHA), romidepsin, entinostat and valproic acid) exhibit anti-tumor effects in a variety of tumors *in vitro* and *in vivo* [15–17]. Vorinostat and romidepsin have been approved by U.S. Food and Drug Administration (FDA) for the treatment of cutaneous T-cell lymphoma [18]. AR-42, a novel hydroxamate-tethered phenylbutyrate derivative, is a potent general HDAC inhibitor with selective cytotoxicity in various human tumor models [19–21]. Compared with other HDAC inhibitors, AR-42 was more potent in inducing apoptosis and suppressing tumor xenograft growth in chronic lymphocytic leukemia (CLL) cell lines [19]. However, the effects of AR-42 in hepatocellular carcinoma have not yet been studied.

In the present study, we confirmed high levels of HDAC5 in tumor tissues, which suggested the poor survival in patients with hepatocellular carcinoma. In addition, we found that high levels of HDAC5 induced proliferation and inhibited apoptosis in HCC cell lines. AR-42 inhibited cancer cell proliferation through the induction of cell apoptosis primarily by targeting HDAC5. These findings indicate that HDAC5 shows promise as a potential therapeutic target and AR-42 may be a new drug candidate for HCC therapy.

## RESULTS

### The inhibitory effect of AR-42 on HCC cell viability

**Figure 1:**
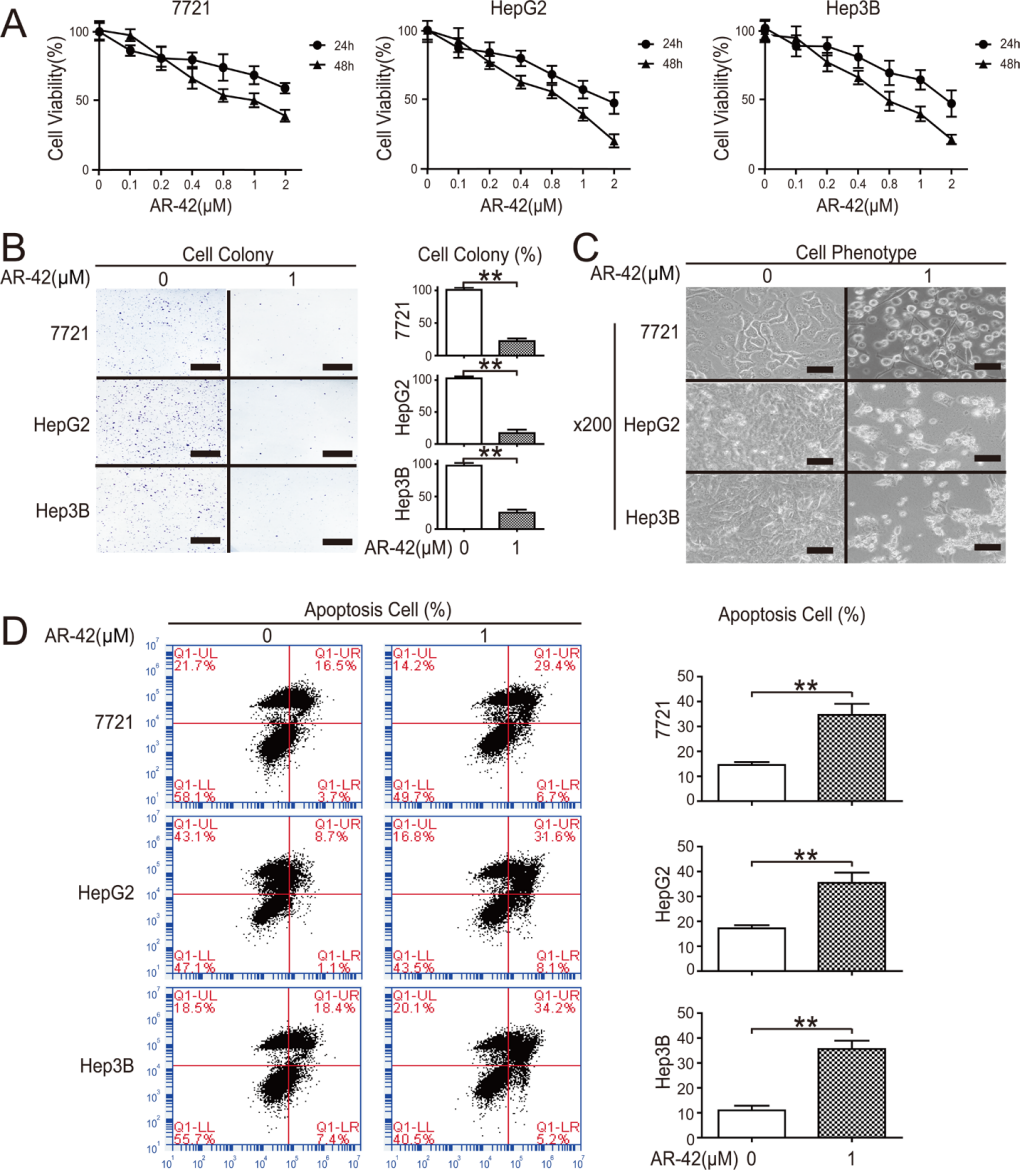
AR-42 reduces HCC cell viability Cells were treated with AR-42 and quantified via CCK-8 assay (**A**). Cells were treated with AR-42. Colonies were stained with crystal violet (left) and quantified (right). Scale bars, 1 cm (**B**). Cells were treated with AR-42 and morphological changes were observed. The magnification is × 200. Scale bars, 200 μm (**C**). Cells were treated with AR-42, subjected to flow cytometry (left) and quantified (right) (**D**). Data represent the mean ± SEM, *n* ≥ 3. *P < 0.05, **P < 0.01.

CCK8 assay results showed that the 50% growth inhibitory concentration (IC50) of AR-42 at 48 h was approximately 0.9 μM in 7721 cells, HepG2 cells and Hep3B cells (Figure 1A). Consistent with CCK8 assays, colony formation in HCC cells was significantly decreased after AR-42 treatment (Figure 1B). Flow cytometry confirmed that AR-42-induced cell death (Figure 1C) was due to apoptosis (Figure 1D). Collectively, these results revealed that AR-42 treatment reduced HCC cell viability.

### HDAC5 overexpression promoted growth in HCC cells

**Figure 2:**
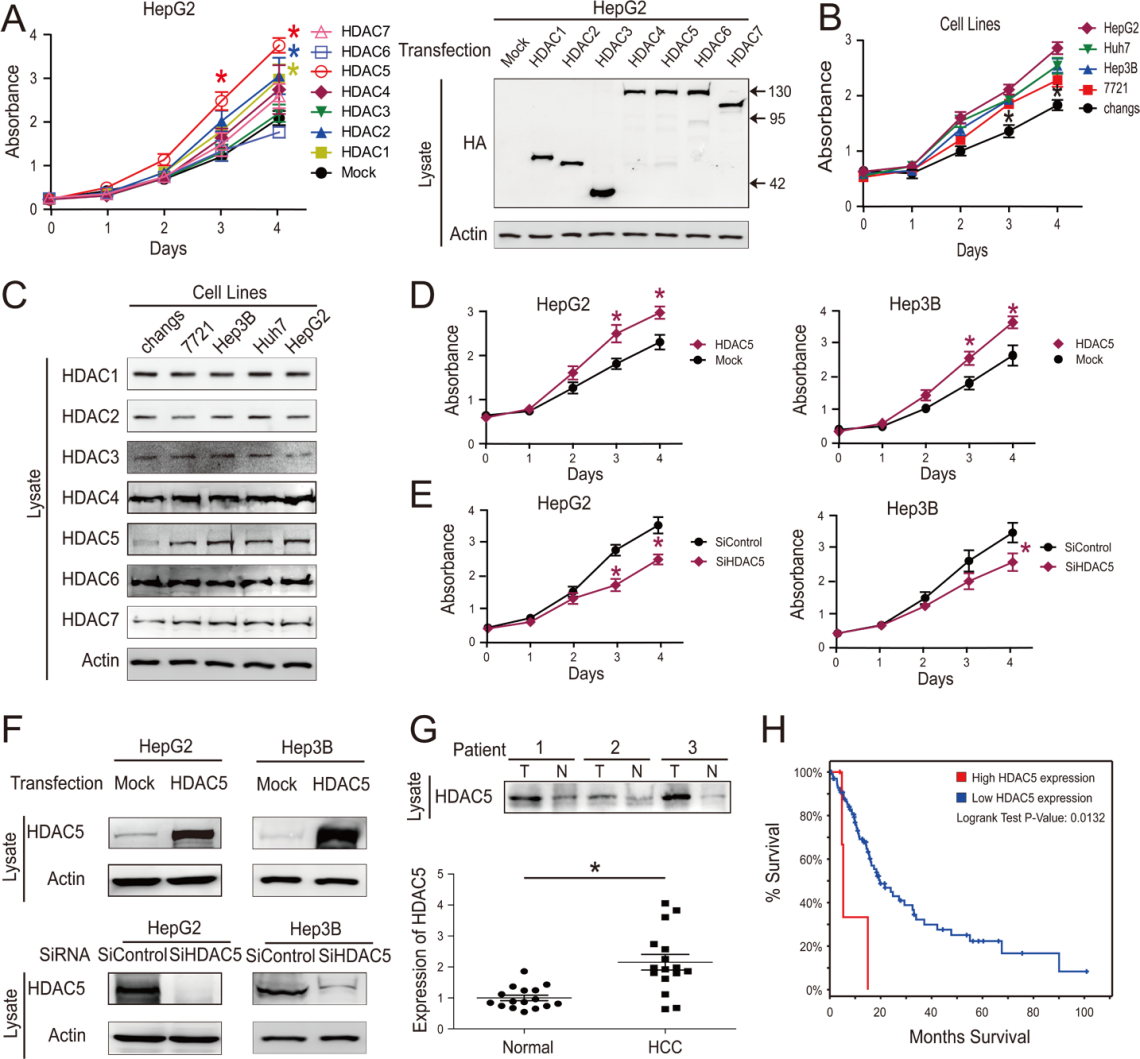
HDAC5 overexpression promotes HCC cell proliferation After transfection with HDAC overexpression vector, cell growth was quantified via CCK-8 assay (left). Transfection were confirmed by western blotting (right) (**A**). Cell growth as quantified via CCK-8 assay (**B**). HDAC protein levels were detected by western blot (**C**). HepG2 and Hep3B cell proliferation was analyzed via CCK8 assay following transfection with HDAC5 overexpression vector (**D**). HepG2 and Hep3B cell proliferation was analyzed via CCK8 assay following siRNA transfection. (**E**) Transfection efficiency was confirmed by western blotting (**F**). HDAC5 protein levels in tumor (T) and adjacent normal tissues (N) from 16 HCC patients were detected (top) and quantified (bottom) (**G**). 5-year survival was reduced for 269 HCC patients with elevated HDAC5 mRNA expression. (**H**) Data represent the mean ± SEM, *n* ≥ 3. *P < 0.05, **P < 0.01, NS not significant.

Certain HDACs (especially HDAC 1, 2, 5 and 6) are upregulated in cancer cells and this correlates with reduced survival in patients with hepatocellular carcinoma [12–14, 22–26]. In some of the same studies, the expression of other HDACs (especially HDAC 4, 9, 10 and 11) did not differ between HCC and noncancerous liver tissue [22, 27]. When we assessed the impacts of HDACs 1–7 on HepG2 cell proliferation, we found that only HDAC5 significantly promoted proliferation (Figure 2A). We evaluated the relationship between cell proliferation and HDAC expression using a normal liver cell line (Chang's) and four human HCC cell lines (7721, Hep3B, Huh7 and HepG2). We found that only HDAC5 protein levels positively correlated with cell proliferation (Figure 2B and 2C). HDAC5 overexpression and knockdown in HCC cell lines also confirmed this proliferation-promoting effect (Figure 2D, 2E and 2F). Moreover, HDAC5 expression was significantly increased in HCC tissues compared with adjacent normal tissues (Figure 2G). We queried The Cancer Genome Atlas (TCGA) database, which contains clinically annotated genomic data from 269 HCC samples [28, 29], and found that HDAC5 mRNA was overexpressed (Z > 2) in 19/269 HCC cases (7%), and was associated with reduced patient survival (*P* = 0.0132, log-rank test) (Figure 2H).

### AR-42 induced HepG2 cell apoptosis *in vitro*

**Figure 3:**
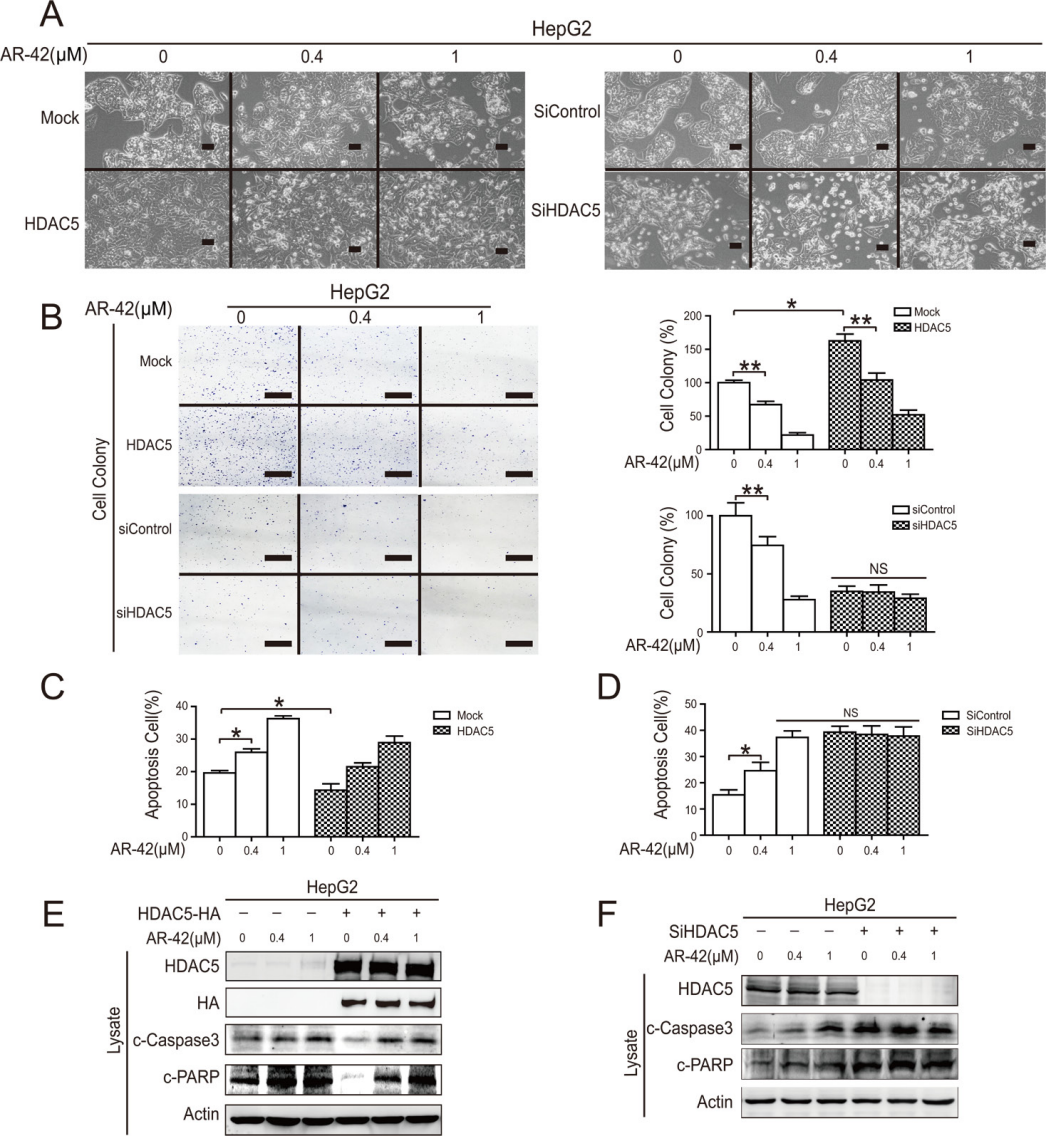
AR-42 induced HepG2 cell apoptosis *in vitro* Cells were transfected with HDAC5 overexpression vector or siRNAs, then treated with AR-42. Morphological changes were observed. The magnification is × 100. Scale bars, 200 μm (**A**). Transfected cells were treated with indicated AR-42. Colonies were stained with crystal violet (left) and quantified (right). Scale bars, 1 cm (**B**). Transfected HepG2 cells were treated with AR-42, and cell apoptosis was analyzed via flow cytometry (**C**). HDAC5-overexpressing cells were treated with AR-42, and cell apoptosis was subjected to flow cytometry and quantified (**D**). HDAC5-overexpressing HepG2 cells were treated with AR-42, and subjected to western blot (**E**). HepG2 cells transfected with HDAC siRNA were treated with AR-42, and subjected to western blot (**F**). Data represent the mean ± SEM, *n* ≥ 3. *P < 0.05, **P < 0.01, NS not significant.

We found that AR-42 increased cell death in a dose-dependent manner in HCC cell lines, and, consistent with previous studies, HDAC5 overexpression partially reversed this effect (Figure 3A) [30]. Transfection of cells with siHDAC5 significantly decreased colony formation and partially blocked AR-42 inhibition of clonogenicity (Figure 3B). Flow cytometry results demonstrated that AR-42 increased the relative amount of cell apoptosis while HDAC5 overexpression protected HepG2 cells against this effect. Conversely, siHDAC5 increased HepG2 cell apoptosis (Figure 3D). AR-42-induced caspase substrate (polyADP ribose polymerase (PARP) and caspase 3) cleavage was mildly reversed by HDAC5 overexpression, and was effectively abrogated by siHDAC5 (Figure 3E and 3F). Collectively, these results indicate that HDAC5 participates in AR-42-induced apoptosis in HepG2 cells and that HDAC5 might be the target of AR-42.

### HDAC5 is the direct target of AR-42

**Figure 4:**
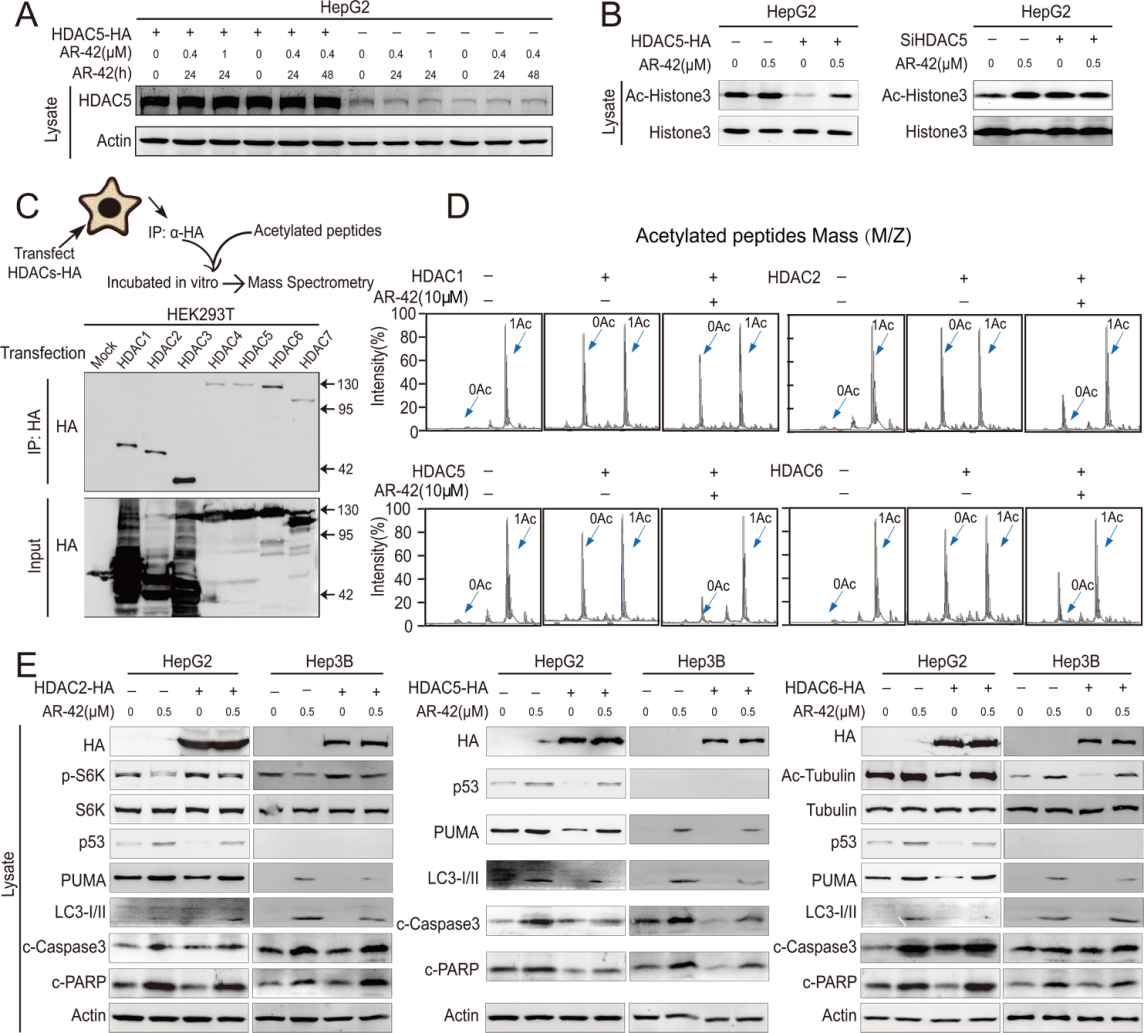
HDAC5 is the direct target of AR-42 HDAC5-overexpressing HepG2 cells were treated with AR-42 and subjected to western blot (**A**). Transfected HepG2 cells were treated with AR-42, and subjected to western blot (**B**). Schematic diagram of the *in vitro* deacetylation assay with HDAC5. The immunoprecipitated protein corresponding to HDACs-HA was subjected to western blot (**C**). The immunoprecipitated protein was incubated with acetylated peptides with or without AR-42, and the rate of deacetylation was determined using Mass Spectrometry (MS) (**D**). HDAC-overexpressing cells were treated with AR-42, and subjected to western blot (**E**). Data represent the Mean ± SEM, *n* ≥ 3. *P < 0.05, **P < 0.01, NS not significant.

To determine whether AR-42 inhibited HDAC5 activity, HepG2 cells were treated with AR-42 following transfection with HDAC5 overexpression vector. We found that HDAC5 protein levels were unchanged by AR-42 treatment (Figure 4A). Inhibition of HDAC5 activity by AR-42 was subsequently assessed by evaluating acetylation of histone H3, the downstream target of HDAC5 [31]. We found that AR-42 increased histone H3 acetylation, and HDAC5 overexpression reversed this effect (Figure 4B). These data suggested that AR-42 inhibits HDAC5 activity.

We then attempted to determine which HDAC was responsible for AR-42-induced apoptosis (Figure 4C). To elucidate the direct target of AR-42, we used an *in vitro* deacetylation system. AR-42 treatment inhibited HDAC 2, 5 and 6 deacetylation activity, but had only a marginal inhibitory effect on HDAC1 (Figure 4D). We investigated the effects of HDACs 2, 5 and 6 on their downstream targets following AR-42 treatment, including acetylated α-Tubulin, phospho-p70 S6 kinase, LC3, P53 and PUMA [11, 24, 26, 32]. We found that only HDAC5 could rescue AR-42-induced cell apoptosis in HCC cell lines (Figure 4E).

### Effect of AR-42 on tumor xenografts

**Figure 5:**
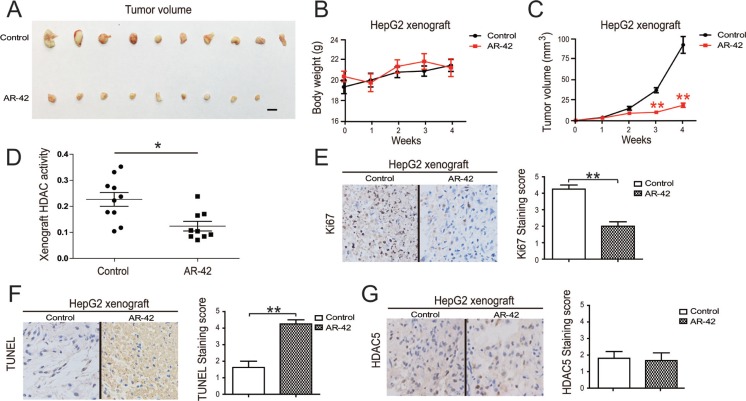
Effect of AR-42 on tumor xenograft models Systemic delivery of AR-42 suppresses HepG2 cell xenograft tumor growth in nude mice. Tumors were photographed after all animals were sacrificed. Scale bars, 1 cm. (**A**) The body weights of tumor-burdened mice (**B**). The xenograft tumor sizes (**C**). Relative activity of HDACs extracted from HepG2 xenograft samples (**D**). Xenograft samples were stained with Ki-67 (left) and staining was quantified (right) (**E**). Xenograft samples were stained with TUNEL (left) and staining was quantified (right) (**F**). Xenograft samples were stained with HDAC5 (left) and staining was quantified (right) (**G**). Data represent the mean ± SEM, *n* ≥ 3. *P < 0.05, **p < 0.01, NS not significant.

We employed a HepG2 cell tumor xenograft model to evaluate the *in vivo* anti-cancer and HDAC inhibitory activity of AR-42, and found that AR-42 administration significantly inhibited tumor growth (Figure 5A and 5C). The body weights of treated mice were used as indicators of health [33]. AR-42 treatment did not affect mouse body weight, indicating that the mice did not experience evident toxicity *in vivo* (Figure 5B). An assessment of activity in HDACs extracted from HepG2 xenograft samples confirmed the inhibitory effect of AR-42 *in vivo* (Figure 5C). Furthermore, histological sections of xenograft samples were stained with TUNEL and Ki-67, markers of cell apoptosis and proliferation, respectively [33]. Consistent with the *in vitro* results, AR-42 administration increased TUNEL staining and reduced Ki-67 staining in xenograft tissues, confirming the anti-tumor effect of AR-42 (Figure 5E and 5F).

HDAC5 phosphorylation liberates nuclear transcription factors through nuclear export of the phosphorylated HDAC5 [34]. We found that HDAC5 mainly localized to the nucleus, but was also observed in the cytoplasm. AR-42 treatment did not significantly change the location and protein level of HDAC5 in HepG2 xenograft samples (Figure 5G). These data collectively demonstrated the apoptosis-inducing and proliferation-inhibiting activity of AR-42 *in vivo*.

## DISCUSSION

HDACs are promising targets for anti-tumor therapeutics, as Class I and II HDACs play important roles in tumorigenesis [4–8]. Numerous reports indicate that HDACs are overexpressed in many cancers and inhibit specific tumor suppressor genes, resulting in aberrant epigenetics in cancer cells compared to adjacent normal tissue [9, 10]. In hepatocellular carcinoma (HCC), some HDACs (especially HDACs 1, 2, 5 and 6) are upregulated in cancer cells compared to normal tissue [12–14, 22–26]. The expression of HDAC isoenzymes correlates with clinicopathological factors, suggesting that these HDACs may be potential indicators of reduced patient survival. However, modifications in HDAC expression (especially HDACs 1, 3, 7 and 8) did not affect HCC cell proliferation [23, 35]. Since HDACs 1, 2, 5 and 6 were differentially expressed and correlate with clinicopathological factors in HCC, we screened the effects of these HDACs on HCC cells proliferation and confirmed that HDAC5 enhances cell proliferation and inhibits apoptosis, indicating that HDAC5 could be the potential target of the chemotherapeutic, AR-42. As AR-42 is a broad HDAC inhibitor, its inhibitory effects on other HDACs besides HDAC5 could not be ignored. Therefore, we evaluated the inhibitory effect of AR-42 to HDACs 1, 2, 5 and 6 by employing mass spectrometry and confirmed AR-42 could directly inhibit HDACs 2, 5 and 6 *in vitro*. At the molecular level, HDAC5 overexpression not only partially reversed the AR-42 induced cell apoptosis, but also reversed the AR-42 induced protein increase of p53 and apoptosis-related proteins, while HDAC2 and HDAC6 did not show a similar effect. Consistent with *in vitro* data, AR-42 significantly inhibited the *in vivo* activity of HDAC5, and induced apoptosis in HepG2 xenograft tissues. Together, these data suggest that AR-42 induces HCC cell apoptosis by inhibiting the activity of HDAC5.

HDAC5 can cause significant modifications in heterochromatin structure that trigger cancer cells to undergo apoptosis [11]. HDAC5 is also regulated by post-translational modification: phosphorylation of HDAC5 by HDAC5 kinases liberates nuclear transcription factors through nuclear export of the phosphorylated HDAC5 [34]. The exit of HDAC5 from the nucleus after prolonged genotoxic stress coincides with TIP60-dependent acetylation of p53 at K120 and recruitment of p53 to the BAX proapoptotic target gene promoter, and with the expression of the proapoptotic target gene [36]. We found that AR-42 treatment did not significantly change the cellular location or protein level of HDAC5 in HepG2 xenograft samples, indicating that AR-42 inhibits the deacetylation activity of HDAC5, rather than its expression.

Acetylation increases p53 protein stability and upon acetylation of p53 at K120, p53 preferentially activates the expression of proapoptotic genes BAX, PUMA, DR5 and NOXA [36]. We evaluated the role of P53 in AR-42-induced apoptosis and found that AR-42 significantly increased protein levels of P53 and activated the expression of the downstream proapoptotic gene PUMA. HDAC5 overexpression not only rescued AR-42-induced cell apoptosis, but also reversed AR-42-induced upregulation of p53 and PUMA in HepG2 cells. However, we observed the same results in Hep3B without p53, which indicated that AR-42 promotes HCC cell apoptosis partially by increasing HDAC5 acetylation of p53.

The present work found that HDAC5, a key regulatory factor for cell proliferation and apoptosis, was associated with poor prognosis in HCC patients. For the first time, our results demonstrated that AR-42 induces cell apoptosis in HCC cells by targeting HDAC5, and therefore shows potential as a new drug candidate for HCC therapy.

## MATERIALS AND METHODS

### Cell culture and reagents

Human liver cell lines (HepG2, Huh-7, Hep3B, 7721 and Chang's) and HEK293 were gifts from the Institute of Biochemistry and Cell Biology, Shanghai Institutes for Biological Sciences, Chinese Academy of Sciences (Shanghai, China) and the Zhao lab of Fudan University (Shanghai, China) [37, 38]. Cells were maintained in Dulbecco's modified Eagle's medium (DMEM) (Invitrogen, Carlsbad, CA, USA) containing 10% fetal bovine serum (Invitrogen), penicillin (Invitrogen) (100 U/ml) and streptomycin (Invitrogen) (100 U/ml). AR-42 (Selleck, Houston, TX, USA) and the HDAC Assay Kit (Millipore, Billerica, MA, USA) were commercially purchased.

### Patient samples

Liver cancer samples were acquired from the Department of Hepatobiliary Surgery, The Eastern Hepatobiliary Surgery Hospital of Second Military Medical University. The use of all patient samples in this study was approved by the Ethics Committee of The Second Military Medical University.

### Immunohistochemistry

Tumor specimens were fixed in 4% formalin and embedded in paraffin. The sections were incubated with TUNEL kit buffer (Gugebio, Wuhan, China) or anti-Ki67 antibodies (Santa Cruz, Dallas, TX, USA) and subsequently with DAPI (Gugebio) and the corresponding secondary antibody (Zsbio, Beijing, China). Sections were then treated with immunoperoxidase using the DAB kit (Zsbio) and scored [39].

### Cell transfection

Cells were transfected using Lipofectamine 2000 (Invitrogen) according to the manufacturer's protocol. The siRNAs were commercially purchased (Santa Cruz) [11]. The control vector and HDAC1-7 expression vectors were kindly provided by the Zhao lab of Fudan University (Shanghai, China).

### Western blotting analysis

Cells were lysed with 0.5% NP40 lysis buffer and proteins were blotted following standard protocol. Signals were probed using the chemiluminescence ECL plus reagent (Thermo, Grand Island, NY, USA) and detected using a Typhoon FLA9500 scanner (GE, Fairfield, CT, USA). Primary antibodies were as follows: HDAC1 (abcam, Cambridge, UK), HDAC2 (abcam), HDAC3 (abcam), HDAC4 (CST, Danvers, MA, USA), HDAC5 (Santa Cruz), HDAC6 (CST), HDAC7 (abcam), cleaved caspase-3 (Antibody Revolution, San Diego, CA, USA), cleaved PARP (CST), β-actin (Sigma), acetyl-histone H3 (Millipore), acetylated α-tubulin (abcam), α-tubulin (CST), phospho-p70 S6 kinase (CST) and p70 S6 kinase (CST). LC3, P53, PUMA, HA, and anti-histone H3 antibodies were kindly provided by the Zhao lab of Fudan University (Shanghai, China).

### Cell viability and clonogenic assay

Cels viability was determined using the CCK-8 colorimetric assay in 96-well plates (2 × 10^3^ cells/well) (Dijindo, Minato-ku, Tokyo, Japan). The absorbance at 450 nm was recorded using a micro-plate reader. For the clonogenic assay, cells were seeded into 6-well plates (5 × 10^2^ cells/well) and cultured for 10 days. Colonies were fixed with 4% paraformaldehyde, stained with crystal violet, and then counted.

### Apoptosis assay

Cell apoptosis was measured by flow cytometry using the AnnexinV-FITC/PI Apoptosis Detection Kit (BD, Franklin Lakes, NJ, USA) following the manufacturer's instructions.

### HDAC deacetylation assay

Cells were lysed in NP-40 buffer containing 50 mM Tris-HCl (pH 7.5) (Sigma, St Louis, MO, USA), 150 mM NaCl (Sangon, Shanghai, China), 0.5% Nonidet P-40 (Sigma), 1 μg/ml aprotinin (Sigma), 1 μg/ml leupeptin (Sigma), 1 μg/ml pepstatin (Sigma), 1 mM Na_3_VO_4_ (Sigma) and 1 mM PMSF (Sigma). For immunoprecipitation, 500 μl of cell lysate was incubated with HA antibody (provided by the Zhao lab of Fudan University) for three hours at 4°C with rotation. Then, 30 μl Protein A Agarose (Millipore) was added for 12 hours at 4°C with rotation, and the beads were washed three times with lysis buffer before proteins were dissolved in loading buffer. Deacetylation assays were carried out in the presence of 5 μg enzyme and 0.3 μg peptide in 30 μl reaction buffer (30 mM HEPES (Sigma), 0.6 mM MgCl_2_ (Sangon), 1 mM DTT (Sigma), 1 mM NAD^+^ (Sigma), 10 mM PMSF (Sigma)). The deacetylation reaction was incubated for 3–5 hours at 37°C before the mixture was desalted by passing it through a C18 ZipTip (Millipore). The desalted samples were analyzed using a MALDI-TOF/TOF mass spectrometer (Applied Biosystems, Grand Island, NY, USA). The acetylated peptide used in the assay was GILRRLKK^Ac^YDNCWL (Glssale, Shanghai, China).

### Liver cancer xenograft model

Nude mice were purchased from the Department of Laboratory Animal Science, Fudan University. HepG2 cells (5 × 10^6^) in FBS-free DMEM were subcutaneously injected into the flanks of mice. Once xenograft tumors were palpable, mice were treated with AR-42 at a dose of 10 mg/kg bodyweight in 100 ml volume via tail vein injection twice a week for three weeks. Tumor volume was calculated using the formula, length (L) × width (W) × height (H) × 0.5236. The Animal Welfare Committee of the Department of Laboratory Animal Science, Fudan University, approved all procedures involving animals.

### Statistics

Data were expressed as means ± standard error of the mean (SE). The data were analyzed through one-way ANOVAs followed by post hoc Duncan tests (SPSS 17.0). *P* < 0.05 was considered significant.
